# The Synergistic Effect of Concomitant Schistosomiasis, Hookworm, and Trichuris Infections on Children's Anemia Burden

**DOI:** 10.1371/journal.pntd.0000245

**Published:** 2008-06-04

**Authors:** Amara E. Ezeamama, Stephen T. McGarvey, Luz P. Acosta, Sally Zierler, Daria L. Manalo, Hai-Wei Wu, Jonathan D. Kurtis, Vincent Mor, Remigio M. Olveda, Jennifer F. Friedman

**Affiliations:** 1 Department of Community Health and International Health Institute, Brown University Providence, Rhode Island, United States of America; 2 Health Effects Institute, Boston, Massachusetts, United States of America; 3 Department of Immunology, Research Institute of Tropical Medicine, Manila, The Philippines; 4 Department of Community Health, Brown University, Providence, Rhode Island, United States of America; 5 Department of Pathogenic Biology, Nanjing Medical University, Nanjing, Jiangsu, China; 6 Center for International Health Research, Rhode Island Hospital, Providence, Rhode Island, United States of America; 7 Department of Pathology and Laboratory Medicine, Brown University Medical School, Providence, Rhode Island, United States of America; 8 Department of Pediatrics, Brown University, Providence, Rhode Island, United States of America; London School of Hygiene & Tropical Medicine, United Kingdom

## Abstract

**Objective:**

To estimate the degree of synergism between helminth species in their combined effects on anemia.

**Methods:**

Quantitative egg counts using the Kato–Katz method were determined for *Ascaris lumbricoides*, hookworm, *Trichuris trichiura*, and *Schistosoma japonicum* in 507 school-age children from helminth-endemic villages in The Philippines. Infection intensity was defined in three categories: uninfected, low, or moderate/high (M+). Anemia was defined as hemoglobin <11 g/dL. Logistic regression models were used to estimate odds ratios (OR), 95% confidence intervals (CI), and synergy index for pairs of concurrent infections.

**Results:**

M+ co-infection of hookworm and *S. japonicum* (OR = 13.2, 95% CI: 3.82–45.5) and of hookworm and *T. trichiura* (OR = 5.34, 95% CI: 1.76–16.2) were associated with higher odds of anemia relative to children without respective M+ co-infections. For co-infections of hookworm and *S. japonicum* and of *T. trichiura* and hookworm, the estimated indices of synergy were 2.9 (95% CI: 1.1–4.6) and 1.4 (95% CI: 0.9–2.0), respectively.

**Conclusion:**

Co-infections of hookworm and either *S. japonicum* or *T. trichiura* were associated with higher levels of anemia than would be expected if the effects of these species had only independent effects on anemia. This suggests that integrated anti-helminthic treatment programs with simultaneous deworming for *S. japonicum* and some geohelminths could yield a greater than additive benefit for reducing anemia in helminth-endemic regions.

## Author Summary

Polyparasitic infections have been recognized as the norm in many tropical developing countries, but the significance of this phenomenon for helminth-associated morbidities is largely unexplored. Earlier studies have suggested that multi-species, low-intensity parasitic infections were associated with higher odds of anemia among school-age children relative to their uninfected counterparts or those with one low-intensity infection. However, specific studies of the nature of interactions between helminth species in the mediation of helminth-associated morbidities are lacking. This study quantifies the extent to which polyparasitic infections have more than the sum of adverse effects associated with individual infections in the context of childhood anemia. This study found that the risk of anemia is amplified beyond the sum of risks for individual infections in children simultaneously exposed to 1) hookworm and schistosomiasis, and 2) hookworm and trichuris, and suggests that combined treatment for some geohelminth species and schistosomiasis could yield greater than additive benefits for the reduction of childhood anemia in helminth-endemic areas. However, more studies to understand the full range of interactions between parasitic species in their joint effects on helminth-associated morbidities will be necessary to better predict the impact of any future public health intervention.

## Introduction

The high prevalence of polyparasitic infections of geohelminth and schistosomiasis infections has received considerable attention in epidemiologic literature [1],[2],[3],[4],[5],[6],[7]. This profile of infection is now recognized to be the norm for many residents of parasite endemic regions [3],[5],[6],[7],[8],[9]. There are few human studies of the morbidity implications of polyparasitism, which may elicit a range of biologic interactions between the host's immune system and the invading parasites [10]. One possible form of interaction is synergism, which implies that the adverse health effect associated with multiple species infection is greater than the sum of adverse effects for individual species. This has been observed in mice co-infected with *Trichinella spiralis* and *Heligmosomoides polygyrus* [11]. The generalizability of observations from murine systems to human populations is unknown. Understanding the morbidity implications of polyparasitism, including any evidence of synergism between helminths in their cumulative health impacts, could provide valuable information for healthcare providers in many developing countries who must decide how aggressively to screen and treat children for polyparasitism in their resource limited settings.

Hookworm infection is causally linked to anemia in humans [12],[13],[14]. Schistosome [15],[16],[17],[18],[19],[20] and moderate or high intensity trichuris [21],[22],[23] infections are also associated with anemia. Further, it is speculated that trichuris infection may exacerbate anemia in the presence of other helminth infections, particularly hookworm [24]. Ascaris infection is known to influence nutritional status [25], but its impact on anemia is less clear. Nevertheless, two epidemiologic studies have found associations between this infection and anemia [26],[27]. The relative contribution of individual helminth species to anemia is governed by different mechanisms for initiating and maintaining anemia. In spite of the real biologic and physiologic differences between species, there is relative consensus that these helminth species contribute in varying degrees to anemia [9],[23],[28],[29],[30],[31],[32].

Contradictions in the epidemiologic literature notwithstanding [33],[34],[35],[36], some human studies have associated polyparasitic infections with higher frequency of malarial attacks, malnutrition and general perception of ill-health in affected individuals [24],[32],[37],[38],[39],[40]. In an earlier study, we found that polyparasitism including *S. japonicum* and geohelminths was associated with higher odds of anemia relative to uninfected or single low intensity infections [10]. However, that study did not formally examine the nature of interactions between parasite species in their effects on anemia. The present study specifically evaluates the degree and direction of biologic interaction between helminth species in their combined effects on anemia. Specifically, we assess whether the odds of anemia in the context of concurrent infections of hookworm, trichuris and *S. japonicum* is significantly higher than would be expected if the species had independent effects on anemia. We hypothesize that individuals with co-infections of hookworm, *S. japonicum,* or trichuris will experience higher anemia burden relative to the total burden of anemia for individuals with single infections.

## Methods

### Study Design

This is a cross-sectional analysis of baseline data from a longitudinal study of immune correlates of resistance to schistosomiasis re-infection conducted in Leyte, The Philippines, among participants between 7 and 30 years old. The study area is endemic for both *S. japonicum* and geohelminths [41],[42],[43],[44]. In total 74.3% (N = 1262/1699) of individuals aged 7–30 years residing in the 3 study villages were screened for the presence of *S. japonicum* infection by duplicate examination of 3 stool samples prior to enrolment. The prevalence of infection with *S. japonicum* in this age range was 60.0%. The study participants and the method of sampling have been described elsewhere[45],[46]. Of the sample screened prior to enrollment, 70.8% (N = 894/1262) were between the ages of 7–18 years. Included in the present study are children between the ages of 7 and 18 years who provided a stool sample, child assent (in addition to informed consent from their parents for participation in the study), and were not pregnant (N = 512). Of these, 5 children lacked information key covariates under investigation and were excluded from the present analyses leaving an effective sample size of 507 children. The institutional review boards of Brown University and The Philippines Research Institute of Tropical Medicine approved this study. The socio-demographic characteristic of the study sample is described in Table 1.

**Table 1:** Socio-demographic and Parasitological Characteristics of Study Sample (N = 507) - Crude Associations with Anemia and Prevalence of Anemia within Co-infection Categories.

	N* (%)	**Odds of Anemia Odds Ratio (95%Confidence Interval)**
Anemia†	101 (19.9)	----
**Age (years)**		
7–12	298 (58.8)	1.84 (1.24–2.68)
13–18	209 (41.2)	1.00
**Male**	289 (57.0)	2.79 (1.77–4.41)
Average Tanner Stage‡ [Mean(SD)]	2.1 (0.9)	0.74 (0.57–0.97)
**Socioeconomic Status**	**N (%)**	
Low	162 (32.0)	2.57 (1.44–4.61)
Medium	171 (33.7)	1.82 (1.04–3.18)
High	174 (34.3)	1.00
Weight for Age Z-score§		
Normal	65 (12.8)	1.00
Mild under-weight	130 (25.6)	2.46 (1.52–3.98)
Severe under-weight	312 (61.5)	4.26 (2.14–8.40)
***S. japonicum***		
None	105 (20.7)	1.00
Low Intensity	272 (53.7)	3.45 (1.54–7.70)
M+ Intensity**	130 (26.6)	6.47 (2.75–15.2)
**Hookworm**		
None	225 (44.6)	1.00
Low Intensity	237 (46.9)	0.76 (0.47–1.22)
M+ Intensity	43 (8.5)	4.40 (2.16–8.94)
***T. trichiura***		
None	36 (7.1)	1.00
Low intensity	218 (43.2)	1.42 (0.61–3.29)
M+ Intensity	251 (49.7)	1.45 (0.59–3.59)
*A. lumbricoides* ††		
None	103 (20.4)	1.00
Low Intensity	117 (23.2)	0.73 (0.39–1.36)
M+ Intensity	285 (56.4)	0.68 (0.40–1.12)
**Joint Distribution of Concomitant infections in Study Sample** ‡‡	**Number of individuals**	**Prevalent Anemia within Categories of Exposure (%)**
**Hookworm & ** ***S. japonicum***		
Reference§§	219	13.70%
Low intensity co-infection of *S. japonicum* & Hookworm	134	14.20%
M+ intensity infection of *S..japonicum* only	116	26.70%
M+ intensity infection of Hookworm only**	28	39.30%
M+ intensity co-infection of *S. japonicum* & Hookworm	15	73.30%
**Hookworm & Trichuris**		
Reference	129	15.50%
Low intensity co-infection of hookworm & trichuris	111	17.40%
M+ intensity infection of Trichuris only	229	19.90%
M+ intensity infection of Hookworm only	19	42.10%
M+ intensity co-infection of Trichuris & Hookworm	24	58.30%
***S. japonicum*** ** & Trichuris**		
Reference	72	9.70%
Low intensity co-infection of *S. japonicum* & trichuris	112	17.70%
M+ intensity infection of Trichuris only	179	16.80%
M+ intensity infection of *S. japonicum* only	57	29.80%
M+ intensity co-infection of *S. japonicum* & Trichuris	74	33.80%

*: Total Sample could differ within strata as a function of missing data.

**†:** Anemia defined as Hemoglobin levels< = 11 g/dL.

**‡:** Tanner stage is a measure of sexual maturity, with higher numbers reflecting greater sexual maturity. Range: 1–4.5.

**§:** Nutritional status defined by Z-scores using American children as reference population based on 1976 NCHS data. Normal = z-score >−1, mild: −2<Z-score< = −1, severe: Z-score<−2.

**: M+ -includes moderate and high intensity infection burdens collapsed into one category.

**††:** The prevalence of anemia by ascaris intensity was 23.8%, 19.5% and 18.8% for low and M+ ascaris intensity infections respectively.

**‡‡:** For any pair of helminths, the category “M+ intensity infection only” includes children infected at M+ intensity with only one of the two species (e.g. S. japonicum) while the other infection is either completely absent or present only at low intensity.

**§§:** The reference group for any pair of helminth co-infections is defined to include the following: a) children uninfected by either species and b) children infected by only 1 of the species at low intensity.

### Infection Intensity

Infection burdens for *S. japonicum*, Hookworm, *T. trichiura*, and *A. lumbricoides*, were determined by duplicate examination of three stool specimens per study participant using the Kato Katz method. The number of eggs per gram (EPG) of stool was used to define three infection intensity levels - uninfected, low, moderate or high (M+), for ascaris, trichuris and *S. japonicum*- using World Health Organization recommended cut-offs [23]. Because consensus cut-offs for hookworm intensity are lacking [47] and evidence that the EPG of hookworm associated with morbidity varies by population, sex and age [47],[48], empirical distributions were used to define low and M+ hookworm infection intensity as 1–999 EPG and > = 1,000 EPG respectively.

### Anemia

Complete hemograms were determined on a Serono Baker 9000 hematology analyzer (Serono Baker Diagnostics, Allentown, PA). Anemia was defined as hemoglobin <11 g/dL [49].

### Primary Determinant: Interaction between Helminth Species

In line with suggestions that interactions of public health relevance ought to be conceptualized as a departure from additive risk profile [50],[51],[52],[53], categorical interaction terms with four levels were defined in this study to assess interactions between hookworm and *S. japonicum,* hookworm and trichuris, *S. japonicum* and trichuris in the mediation of anemia. Of note we attempted to evaluate the interaction between M+ ascaris and each of the three helminth species (hookworm, trichuris and *S. japonicum*) as part of this study. Ascaris infection showed a non-statistically significant trend of protective effect on anemia in exploratory analyses. This pattern was maintained in preliminary analyses of this parasite's interaction with other helminth species. We decided to explore this unexpected finding further as part of a separate ongoing analysis and its results will be presented elsewhere.

For any two helminth species, the levels (categories) of the interaction term differ based on the presence or absence of M+ intensity infections. The reference category consisted of children with no infection or a single low infection. As an example, the 4-level interaction variable for hookworm and *S. japonicum* was defined as follows:

1. **Reference group:** includes children a) free of both hookworm and *S. japonicum* infections, b) with low intensity infection *S. japonicum* only, and c) with low intensity hookworm only.
2. **Moderate** ***S. japonicum*** **only** ***:*** Includes children with a) M+ intensity *S. japonicum* without hookworm, and b) M+ intensity *S. japonicum* with low intensity hookworm infection.
3. **Moderate Hookworm only:** includes children with a) M+ intensity hookworm without schistosomiasis and b) M+ intensity hookworm with low intensity *S*. *japonicum* infection.
4. **Moderate** ***S. japonicum*** **and Hookworm:** children infected at M+ intensity by both *S. japonicum* and hookworm.

There were two reasons for examining interactions at M+ intensity. First, examining interactions at M+ intensity is consistent with the relationship of intensity to adverse health effects [13],[26],[29],[54],[55],[56]. Secondly, stronger interactions were expected between species for individuals multiply exposed to M+ intensity infections. A prior study in this sample found low intensity co-infections were associated with anemia [10]. In light of this, individuals with concomitant low intensity infections were excluded from the reference (lowest risk) group for any pair of helminth co-infection.

### Confounders

Potential confounders of the relationship between anemia and helminth infection were defined in light of known confounders of this association based on published literature [45],[54],[57],[58]. The risk and intensity of helminth infection is known to vary by age, sex, sexual maturity, socioeconomic status (SES) and nutritional status. These factors are simultaneously predictive of the risk of anemia and were considered potential confounders to be analytically adjusted in multivariable analyses.

SES was measured through a detailed questionnaire designed to measure capture the contribution of educational status, social class position, material wealth, and a composite SES. Scores for each of the domains were calculated using principal components analysis to appropriately weight questionnaire items as described by Filmer [59] to derive a summary SES score. For analytic purposes, 3 categories of SES were defined based on the tertiles of the SES distribution. Sexual maturity was defined based on Tanner staging of pubertal development (breasts in girls and genitalia in boys). Staging was based assessment by two physicians in which scores ranging from 1 (pre-pubertal) to 5 (adult) were assigned following standard criteria [60]. For analytic purposes, sexual maturity was dichotomized into low (Tanner score < = 3) and high (Tanner score >3). Nutritional status was assessed by weight-for-age z-score (WAZ) calculated using the National Center for Health Statistics year 2000 reference values in EpiInfo software (version 2000, Atlanta, Georgia). Severe malnutrition was defined as WAZ< = −2 and mild malnutrition was defined by −2<WAZ< = −1. Concomitant infection with helminth species not included in the interaction term could be competing risk factors for anemia, hence the intensity of these species were adjusted for in all multivariable analyses.

### Statistical Methods

Statistical analyses were performed in SAS version 8.1 and included univariate, bivariate and multivariable analyses using logistic regression models. All multivariable analyses were clustered by household residence to allow for the expected dependence of observations within this unit [37],[61],[62] in the estimation of effects using generalized estimating equation (GEE) models with exchangeable correlation matrix structure for dichotomous variables via SAS PROC GENMOD. The odds of anemia were obtained for 3 categories of an interaction variable relative to its reference category. Effective sample size for multivariable models varied from a low of 378 to a high of 401 according to the specific pair interactions under investigation due to the exclusion of concomitant low intensity infections. 95% confidence intervals (CI) were estimated to show the range of values consistent with reported odds ratio (OR) point estimates.

Formal tests of interaction can be directly conducted in cohort studies using the well-described interaction contrast directly estimated using risk ratios from additive models. This parameter is not directly estimable based on outputs from multiplicative models reporting odds ratio as the estimates of effect; however it can be indirectly estimated using the synergy index (SI) and attributable proportion (AP) [63]. SI estimates the proportion of anemia among concurrently infected individuals that is due to the non-additive interaction between co-infecting species and AP estimates the fraction of excess anemia among children concurrently infected by two helminth species due to the non-additive interaction between them. Specific studies have demonstrated that SI [64] and AP [63] are two of the most stable measures of interaction estimable using relative measures derived from logistic regression models. The SI- which theoretically can range from negative infinity to positive infinity - was estimated separately for each pair of helminth species using the following formula: SI = (OR_Infection AB_−1)/[(OR_Infection A_−1)+(OR_Infection B_−1)] where A & B each refers to any two of the three helminth species (hookworm, *S. japonicum*, and trichuris) under evaluation. 95% confidence intervals were estimated for the SI using methods described by Rothman to derive appropriate standard errors for CI estimation [50]. If concomitant infection of any two helminth species had only additive effect on the odds of anemia, then SI = 1 and AP = 0. Synergistic interactions are indicated when SI>1 and AP>0 whereas antagonistic interactions are indicated when SI<1 and AP<0 [50],[51].

To the best of our knowledge, there is currently no established and easily accessible method of testing for the goodness of model fit for the quasi-likelihood based GEE model [65],[66],[67]. Yet understanding model fit and evaluating the contribution of interaction terms to the explanatory capacity of the regression model is critically important for studies investigating public health relevant interactions. In the absence of well established and accessible methods in GEE based models, the goodness of fit was assessed for multivariable models in this study using the Hosmer and Lemeshow goodness of fit test [68],[69] obtained from non-clustered logistic regression models using SAS PROC LOGISTIC. Likelihood ratio tests were also conducted in the same model to evaluate the contribution of interaction terms to the explaining the variability of anemia in the study sample. To the extent that these statistics are not directly assessable in the GEE models the estimates from PROC LOGISTIC models are provided as a guide only. These models were deemed to have appropriate fit if the null hypothesis of adequate model fit was not rejected at the α = 0.05 level and the p-value for the Hosmer-Lemeshow test statistic was > = 0.20. The p-value for the Hosmer-Lemeshow statistic was arbitrarily set at 0.20 for the present analyses to make the statistic more conservative by increasing the rejection error.

## Results

Prevalent malnutrition and anemia in the study sample were 87.1% and 19.9% respectively. The prevalence of infection by individual helminth species in this sample ranged from a low of 55% for hookworm to a high of 93% for trichuris. Only 0.7% of the sample was free of infection by all four parasitic species. 7.4% and 14.3% of children were infected by only one and two helminth species respectively while the majority of the sample (77.6%) were infected by 3 or 4 parasites concurrently (data not shown). Only seven children were simultaneously uninfected by hookworm, *S. japonicum* and trichuris in the analytic sample; all seven children were free of anemia. Among the subset of children uninfected by *S. japonicum* and who were either free, or at most had a low infection, of both hookworm and trichuris (N = 50), the prevalence of anemia was 8% (data not shown). Relative to the reference in each exposure category, anemia was higher among children concurrently infected by any two species regardless of whether co-infection occurred at low or M+ intensity (Table 1). Anemia was highest among children with M+ intensity hookworm or *S. japonicum* infection, especially when these infections were contemporaneous with one another or trichuris. These trends are reinforced in bivariate analyses.

The effect of M+ infections (singly or two M+ together) on anemia and the extent to which the estimated effects for double M+ infections deviate from risk additivity is evaluated for each interaction with adjustment for ascaris intensity, nutritional status, SES, sex, age and other confounders. For each pair of helminths- e.g. hookworm and *S. japonicum*, the odds of anemia are estimated for children with only one of these infections at M+ intensity and for the children infected with the two species together at M+ intensity relative to reference group (children without either infection at M+ intensity).

For hookworm and *S. japonicum* interaction term, single M+ intensity infections of each species were independently and significantly associated with higher odds of anemia relative to the reference group. Among children concomitantly infected by both species at M+ intensity, the odds of anemia was 4 to 7 times higher than that among children infected by hookworm alone or *S. japonicum* alone. The proportion of excess anemia among children concomitantly infected that is due to interactions between co-infecting species was estimated to be at least 60% (Table 2). In addition, the magnitude of association (odds ratio) between concomitant infection and anemia observed among children with both hookworm and *S. japonicum* was three times higher than the magnitude of association expected if both species had independent effects on anemia (data not shown).

**Table 2:** Individual and Polyparasitic Helminth Infections as Determinants of Anemia in School-Age Children with Formal Assessment of Departures from Additive Risk Profile.

Effect of Individual Helminth species on Anemia from Multivariable Logistic Regression Analyses	
	Odds Ratio (95% Confidence Interval)†
**Hookworm**	
None	1.00
Low intensity	0.72 (0.42–1.23)
M+ intensity	4.32 (1.62–11.52)
***S. japonicum***	
None	1.00
Low intensity	3.20 (1.31–7.81)
M+ intensity	5.23 (2.00–13.7)
**Trichuris**	
None	1.00
Low intensity	1.20 (0.43–3.38)
M+ intensity	1.00 (0.31–3.23)
***A. lumbricoides***	
None	1.00
Low intensity	0.72 (0.38–1.67)
M+ intensity	0.45 (0.21–0.86)

	ODDS OF ANEMIA WITHIN CATEGORIES OF INTERACTION	MEASURES OF INTERACTION
INTERACTION CATEGORIES‡	Odds Ratio (95% Confidence Interval)	A) Attributable Proportion (AP)§B) Synergy Index (95% Confidence Interval)**
**Hookworm & ** ***S. japonicum*** **^γ^**		
Reference (N = 219)††	1.00	
M+ intensity infection of S.japonicum only (N = 116)	1.88 (1.06–3.33)	AP = 0.602
M+ intensity infection of Hookworm only (N = 28)	4.37 (1.59–11.96)	SI = 2.87 (1.12–4.62)
M+ co-infection of *S. japonicum* & Hookworm (N = 15)	13.19 (3.82–45.5)	
**Hookworm & Trichuris^γ^**		
Reference (N = 129)	1.00	
M+ intensity infection of Trichuris only (N = 229)	0.97 (0.49–1.92)	AP = 0.22
M+ intensity infection of Hookworm only (N = 19)	4.20 (1.23–14.35)	SI = 1.37 (0.92–2.01)
M+ intensity co-infection of Trichuris & Hookworm (N = 24)	5.34 (1.76–16.21)	
***S. japonicum*** ** & Trichuris^γ^**		
Reference (N = 72)	1.00	
M+ intensity infection of Trichuris only (N = 179)	1.22 (0.48–3.17)	AP = −0.15
M+ intensity infection of S. japonicum only (N = 57)	2.59 (0.97–1.93)	SI = 0.80 (0.32–1.72)
M+ intensity co-infection of S. japonicum & Trichuris (N = 74)	2.44 (0.87–6.78)	
**Model Goodness of Fit Assessment: Hosmer and Lemeshow Goodness of fit test**		
For all multivariable models, χ^2^_8df_ ranged from 4.3213–10.0; P-values: 0.8270–0.2591.		

Analyses are based on multivariable logistic regression models clustered by household of residence and adjusted for the following confounders: age, sex, socioeconomic status, village of residence, nutritional status and tanner stage.

**†:** The estimates below are from logistic regression models that adjusted for the intensity of the 3 other parasitic helminth species (each as an ordinal covariate).

**‡:** These analyses exclude concomitant low intensity infections for any two helminth species under investigation. Sample size varied from 378 to 451 in multivariable analyses as a result of the exclusion of concomitant low intensity infections. For any pair of infections being evaluated, the intensity of the other 2 helminth species are controlled for in regression models. For example, for regression models examining the effect of hookworm and S. japonicum co-infection, ascaris and trichuris intensity are included in regression models as ordinal covariates.

**§:** AP = Attributable Proportion.

**: SI = synergy index. 95% Confidence Interval estimated as described by Kenneth J. Rothman.

γ The interaction variables significantly improved the capacity of the regression model to explain the variability in anemia on the basis of likelihood ratio tests. All *χ* ^2^ **_3_**_df_ ranged from 25.6 to 9.6558; associated p-values ranged from <0.0001 to 0.0217.

**††:** The reference population for any pair of interactions is defined to include the following: a) children uninfected by either species and b) children infected by only 1 of the species at low intensity.

For hookworm and trichuris interaction term, M+ trichuris infection alone was not associated with significantly elevated odds of anemia relative to the reference group; however, hookworm infection alone, and concurrent infection by both hookworm and trichuris were each associated with elevated odds of anemia relative to the reference group. SI indicates the presence of modest synergy for concurrent infection by trichuris and hookworm–although this finding did not achieve statistical significance with the associated 95%CI including the null value. In addition, 22% of prevalent anemia among children with concurrent infections of these species was attributable to the non-additive interaction between them (Table 1).

For terms evaluating interactions between *S. japonicum* and trichuris, M+ Trichuris infection by itself had no impact on anemia status relative to the reference group (Table 2). M+ intensity *S. japonicum* infection alone and concomitant M+ infections of *S. japonicum* and trichuris infections were associated with elevated odds of anemia. The magnitude of association with anemia among children concurrently infected by *S. japonicum* and trichuris, however, did not depart significantly from additive risk profile suggesting that the joint effect of these infections on anemia was similar to the sum of their independent effects on anemia.

The odds of anemia were significantly higher for boys, younger and malnourished children (data not shown). Low intensity ascaris infection was not associated with anemia. Unexpectedly, M+ intensity ascaris infection was associated with lower, rather than higher, odds of prevalent anemia in the study sample.

## Discussion

Despite the reported high prevalence of polyparasitic helminth infections [3],[5],[6],[7],[8],[9], little is known about how these contemporaneous infections interact biologically and influence morbidity. We have examined interactions, at moderate or high intensity, for concomitant infections of three pairs of helminth infections: hookworm and *S. japonicum*, hookworm and trichuris, *S. japonicum* and trichuris. Our results suggest the presence of synergistic interactions for the following pairs of helminths: 1) hookworm and *S. japonicum*, and 2) hookworm and trichuris. Among children co-infected with hookworm and *S. japonicum*, an estimated 60% of the anemia is attributable to the biologic interaction between them. Similarly, with M+ co-infection of hookworm and trichuris, an estimated 22% of the observed odds of anemia were attributable to the synergistic interaction between these species. We found no evidence of departure from additive risk model additivity for co-infection of trichuris and *S. japonicum.* Also found, though not expected, was the protective association between M+ ascaris infection and anemia.

Consistent with our hypothesis, we found that individual helminth species contributed to anemia in different degrees. As in previous studies, we found that hookworm and *S. japonicum* infections were independent risk factors for anemia [16],[20],[30],[70],[71],[72]. We did not find M+ intensity trichuris infection alone to be significantly associated with anemia in this study; however, there was an elevated odds of anemia among children co-infected with trichuris and hookworm.

The link between hookworm and anemia is well known and the mechanism of effect has been described [13],[29],[30],[55],[72],[73],[74],[75],[76]. The mechanisms underlying *S. japonicum* associated anemia are likely multi-factorial [77] including, blood loss in the stool as eggs extravasate through the bowel wall, adult worm ingestion of host red blood cells [78], pro-inflammatory cytokine activity mediating anemia of inflammation whereby bio-available forms of iron are shunted to less bio-available storage forms (ferritin) [79],[80],[81], auto-immune hemolysis [82],[83], and sequestration of red blood cells in an enlarged spleen [84],[85]. High intensity trichuris infection affects iron status through blood loss in the stool, particularly if dysentery syndrome is present. It is hypothesized that trichuris infection could amplify iron deficiency anemia in the presence of hookworm [23],[24]. It has been suggested that ascaris could contribute to anemia by impeding iron absorption in the lumen of the small intestine [56]. This mechanism of action, however, has been investigated by one study and their findings fail to substantiate the hypothesis of iron mal-absorption with ascaris infection [86]. The finding that M+ intensity ascaris infection was inversely associated with anemia was unexpected and persisted in all multivariable models. This unexpected association and detailed assessment of interactions between ascaris infection and the three helminth species: hookworm, *S. japonicum* and trichuris, is the subject of a specific future investigation by our group.

That the odds of anemia was highest in the presence of hookworm and *S. japonicum* infections of M+ intensity is consistent with the biology of these helminth species individually. The much higher odds of prevalent anemia observed in the presence of both helminth species is expected based on the presence of at least two pathways of anemia induction in concurrently infected individuals: active blood loss in the stool and anemia of inflammation [81]. The mechanism responsible for the increased odds above additivity, however, is more complex. *S. japonicum* is known to be associated with pro-inflammatory cytokine elaboration in the human host [87],[88],[89]. While Th2 responses to hookworm antigens are well described [90], a study conducted in Brazil found elevated spontaneous cellular secretion of tumor necrosis factor alpha, a pro-inflammatory cytokine, and interleukin (IL)-10 in individuals with patent hookworm infections [91]. The same study reported decreased spontaneous production of IL-5, a T-helper 2 (TH2) cytokine in these patients. Two other studies conclude that though TH2 responses predominate in the context of hookworm infection, detectable levels of the TH1 cytokines–interferon gamma [90],[92] and IL-12 [93], were also observed in response to antigen stimulation of cell supernatants from hookworm infected individuals. On the basis of these data, we hypothesize that the marked increase in anemia among individuals co-infected with hookworm and *S. japonicum* may be mediated by an enhanced production of pro-inflammatory cytokines. Pro-inflammatory cytokines, particularly IL-6, cause anemia of inflammation by inducing hepatocytes to produce hepcidin, an iron regulatory peptide [94],[95],[96].

A recent study from Brazil demonstrated that there was synergism in the aggregation of hookworm and some helminth species including *S. mansoni* among residents in their study area; however, the impact of this phenomenon on morbidity was not explored [97]. To the best of our knowledge, our report this is the first study to formally assess and report synergistic interactions between *S. japonicum* and hookworm or hookworm and *T. trichiura* in anemia, as a departure from additive risk profile. The demonstrated synergy between these helminths in the context of anemia have, however, been hypothesized by a few investigators [23],[98]. Despite differences in assessment methods that preclude evaluation of the extent to which the adverse health effect of polyparasitic infections departed from additivity of risks, two studies provide some support for our findings. One study reported lower mean hemoglobin among children co-infected with hookworm and trichuris relative to the reference group consisting of children without either infection or children with single infections of either species only [24]. Another study indirectly corroborates our findings by reporting evidence of altered immune function in multiply infected individuals [99]. Specifically, altered immune responses to antigenic preparations were reported for co-infections including *S. mansoni*, hookworm and ascaris. These altered immune responses, the investigators suggest, could impede immune responses to infection [99].

### Strengths and Limitations

Of the few studies that have attempted to link polyparasitic helminth infections to morbidity, all have defined concomitant infections as present or absent for pairs of species [38],[100]. Infection categories defined as such–even when single and combined infection categories are present, often lack a fourth constant reference group without both pairs of infections - make any inferences of biologically and public health relevant interactions difficult [38],[101],[102],[103],[104]. In addition, the majority of these studies were conducted in the context of malaria [101],[102],[103],[104], many did not assess the intensity of co-infecting species, and none of them examined interactions as departure from additivity of risks. We have examined interactions between helminth species in a naturally occurring context for an important morbidity free of possible confounding from malaria while incorporating information on infection intensity. To ensure the most de-confounded estimates of effect possible, important confounders have been adjusted for and the lack of observation independence within household units was analytically addressed. All assessments were made within in a causally relevant framework, allowing some inferences regarding the public health impact of concomitant infections to be made for the sub-group of individuals exposed to multiple helminth species at moderate or higher intensity.

Despite these strengths, we cannot exclude the possibility that anemia predisposes children to multiple infections based on the cross-sectional design of this study, or that unmeasured factors or residual confounding could still bias our results despite careful control for known confounders. Evidence from prospective [57],[105] and randomized clinical studies [42],[100] that helminth infections cause anemia suggests that reverse causality is an unlikely alternative explanation for our findings. Yet, the evaluation of interactions at M+ intensity and the over-sampling of those with *S*. *japonicum* infection as a function of the sampling scheme in the main study, may limit the generalizability of these findings to children co-infected at low intensity, and to representative community samples with different joint distributions of helminth species. Further, the fact that a smaller number of individuals were co-exposed at M+ intensity in this study is likely to have limited the power of this study - potentially making it difficult to achieve statistical significance where one existed for some pairs of helminth species.

### Conclusion

Our study suggests that for children in helminth endemic regions, contemporaneous infections with hookworm and either *S. japonicum* or trichuris may result in higher levels of anemia than would be expected if the effects of these species on anemia were simply additive. The results provide support for the hypothesis that co-infecting parasites, even living in separate anatomic locations, can interact synergistically to modify anemia, likely via the hosts' immune response to concomitant infections. The presence of synergistic interactions between some helminth species as demonstrated here has implications for treatment given the high burden of anemia associated with concomitant infections of hookworm and *S. japonicum* or trichuris. Specifically, our results support the notion that concomitant treatment for *S. japonicum* and some geohelminths may provide an even greater public health benefit than that based on the assumption of additive morbidity. This is a particularly appealing option given joint treatment with albendazole and praziquantel has been shown to significantly improve hemoglobin levels, without greater side-effects than treatment with praziquantel alone [106]. We note however, that in light of the limited number of studies on the morbidity consequence of polyparasitic helminth infections in general, and of the biologic interactions between concurrent infections in the mediation of helminth-associated morbidities in particular, more epidemiologic studies of this phenomenon are necessary to better predict the impact of any public health intervention. Specifically, epidemiologic studies that explore the full range of biologic interactions between co-infecting species - including the possibility of antagonism, in their joint effects on anemia and other helminth-associated morbidities are necessary to fully evaluate the possible risks and benefits of combined mass therapy in helminth endemic regions.
